# Prolonged theoretical classes impact students’ perceptions: an observational study

**DOI:** 10.3389/fpsyg.2024.1278396

**Published:** 2024-02-21

**Authors:** Petra Kotnik, Bart Roelands, Špela Bogataj

**Affiliations:** ^1^Faculty of Health Sciences, University of Novo Mesto, Novo Mesto, Slovenia; ^2^Human Physiology and Sports Physiotherapy Research Group, Vrije Universiteit Brussel, Brussels, Belgium; ^3^Department of Nephrology, University Medical Centre Ljubljana, Ljubljana, Slovenia; ^4^Faculty of Sport, University of Ljubljana, Ljubljana, Slovenia

**Keywords:** mental fatigue, theoretical classes, university students, boredom, sleepiness

## Abstract

Mental fatigue (MF) arises during prolonged demanding cognitive activity and results in acute feelings of tiredness and a decreased physical and/or cognitive performance capacity. An often-overlooked population that is significantly at risk for the development of MF are university students. The current study investigated the impact of prolonged in-person theoretical classes on the perceptions of MF, boredom, and sleepiness among 27 Slovenian university students (first-year physiotherapy). Their subjective experiences at various time points during a 4-h class interspersed with a 20 min break were assessed with a repeated measures ANOVA and consequent Bonferroni post-hoc tests (significance set at <0.05). Subjective MF and sleepiness significantly increased during the first and the second part of the class (*p* < 0.05), while they significantly decreased during the break (*p* < 0.05). Boredom levels only increased significantly during the second part of the class (*p* < 0.05). Additionally, students who had inadequate sleep the night before the class reported higher levels of MF at the beginning of the class. This study highlighted a significant impact of a theoretical class on subjective feelings of mental fatigue and showed that a break in the middle of the class temporarily alleviated this negative impact. These results emphasize the importance of adequate sleep, effective breaks, and strategies to manage cognitive workload in optimizing students’ cognitive well-being and academic performance. Further research is needed to better understand the underlying factors and develop targeted interventions to support students’ cognitive functioning and well-being during prolonged academic sessions.

## 1 Introduction

Mental fatigue (MF) can be defined as a psychobiological state that arises during prolonged demanding cognitive activity and results in acute feelings of tiredness and/or decreased physical and/or cognitive performance capacity (Habay et al., 2021b). Various activities such as intensive studying, problem-solving, activities requiring prolonged attention or professional activities without adequate rest or recovery can trigger the onset of MF. Because of its high prevalence, a multitude of studies have assessed how MF can impact different kinds of human performance (Habay et al., 2023). These studies have mainly shown that MF negatively affects important aspects of cognitive (i.e., accuracy and/or reaction time) (Van Cutsem et al., 2017), physical (i.e., endurance performance) (Van Cutsem et al., 2017; McMorris et al., 2018) and/or technical performance (i.e., sport-specific psychomotor performance) (Habay et al., 2021a). The impact on functional performance also induces greater risks of error in the work place, sports and even during leisure time. This can have further consequences as it might lead to an elevated risk for accidents, injuries and/or incidents (Proost et al., 2022) and it can have profound effects on an individual’s productivity and well-being (Boksem and Tops, 2008). Common examples of professions at risk are drivers, air traffic controllers, medical doctors, but also industry workers (Swaen et al., 2003; Dembe et al., 2005; McCormick et al., 2012). Another – often overlooked - population that is significantly at risk for the development of MF are students.

Indeed, students – certainly in a higher academic context - are often confronted with a high cognitive load as they are required to engage in prolonged, highly cognitively demanding tasks and classes. Sievertsen et al. (2016) performed a study in Danish children and early adolescents and indicated that for every hour later on the day, test scores further declined. At the same time the authors also reported that taking a 20–30 min break improved test scores. Besides that, students often multitask within their academical career, but also with personal responsibilities and hobbies. The continuous switching between these tasks can further aggravate MF. Students are often confronted with deadlines for tasks or presentations, with exams and other assignments that induce increased stress levels that might cause increased levels of MF (Doerr et al., 2015). Additionally, the engagement of students, their drive and motivation to obtain high scores, exam anxiety and other related factors might also induce MF (Herlambang et al., 2021). Despite this being a topic of high societal relevance, it is surprising how little data is available on the fatigue-inducing effect of classes in academia.

The recent COVID pandemic has however triggered research, albeit mainly in an online context. Indeed, according to UNESCO over a billion children worldwide were impacted by sudden school closures because of the pandemic (Ortega Pacheco and Barrero Toncel, 2022). Several studies have been carried out to assess the burden and consequences of following classes online. As an example, Sawant et al. (2023) performed a study to identify the perceptions of medical students (*n* = 346) on online teaching during the COVID pandemic. Two thirds of the students indicated that online classes are less enjoyable than in-person classes, and 71% indicated digital fatigue and a lack of motivation to study (Sawant et al., 2023).

Even though more studies on MF perceptions during classes are emerging, most focus is oriented toward online teaching, while neglecting the in-person theoretical classes. Therefore, the aim of the present study was to assess the MF, boredom and sleepiness perceptions of Slovenian university students during a 4-h in-person (theoretical) class interspersed by a single 20 min break. As secondary objectives we aimed to correlate the feelings of MF at the start of the class with the hours of sleep the night before the class, and we wanted to determine the load that each part of the class (before and after the break) imposed on the students. We hypothesized that following a theoretical class would increase the MF, boredom and sleepiness perceptions, with the break acting as a temporary countermeasure of these subjective feelings.

## 2 Methods

### 2.1 Participants

Participants were 1st year students of the physiotherapy degree, University of Novo mesto, Faculty of Health Sciences, Slovenia. Inclusion criteria were being a 1st year university student, speaking Slovenian fluently, age 18–30 years. Twenty-seven students (11 males and 16 females; 21.4 ± 2.5 years, 172 ± 9.3 cm, 65 ± 13.7 kg) volunteered to participate in the study. Approval for the study was obtained from the Ethics Committee for Research Involving Human Subjects at the Faculty of Health Sciences, University of Novo mesto (UNM 141/2023). Prior to participation, all individuals provided written informed consent. Participants did not receive any form of compensation for their involvement. Data collection took place during scheduled class time.

### 2.2 Study design and procedures

We conducted a study using an observational research approach. Data collection took place during the summer term (April to June) 2023 at the University of Novo mesto Faculty of Health Sciences. Participants were recruited via verbal and written notices. The lecturer, who is a member of the research team, explained the study to all potential participants on the first day of the lectures. Before the start of the data collection a definition of mental fatigue was provided and the participants completed a demographic questionnaire where they were asked about their age, gender, height, weight, sport participation, performance level, volume per week and years of experience. After this they were familiarized with the questionnaires and scales that were used as outcome measures. The classes were conducted between 4:00 PM and 8:00 PM, with data collection taking place during eight sessions. Before the start of each class a pretest checklist was evaluated to check for any abnormalities which warranted exclusion (duration of sleep, sleep quality and caffeine intake) and to obtain additional information on their engagements before the class (work, school, physical activity).

The classes selected were all theoretical classes and their topics ranged from motor development to kinesiotherapy. Outcome assessments were performed at the beginning of each class, after the first part of lectures (prior to the break), after a 20 min break, and at the conclusion of the second part of lectures. Students that left before the class was over were excluded from further analysis. Each part of the lecture lasted for 1 h and 50 min.

### 2.3 Outcome assessments

The subjective feeling of mental fatigue was measured using a visual analog scale (VAS), assessing how mentally fatigued the participants were feeling at that specific moment (M-VAS), and ranged from “Not at all mentally fatigued” to “Extremely mentally fatigued” (Habay et al., 2021a). The subjective feeling of Sleepiness was assessed using a VAS-scale (S-VAS) asking “How are you feeling now?” where participants had to indicate this on a line going from “Extreme alert” to “Extreme sleepy, impossible to stay awake” (Lee et al., 1991). Boredom was also assessed using a VAS-scale (B-VAS) asking “How boring did you find this task?” in which participants had to indicate the feeling of boredom on a scale ranging from “Totally not” to “Extremely boring” (Milyavskaya et al., 2019). All VAS-scales were assessed at four time points: at the beginning of the class (T1), before the break (T2), after the break (T3) and at the end of the class (T4). On the VAS scales, the participants were required to indicate their feeling on a 10-cm horizontal line. The validity and reliability of a visual analog scale to assess fatigue was demonstrated by Lee et al. (1991) and Smith et al. (2019).

Subjective workload of the previous task was measured with The National Aeronautics and Space Administration Task Load Index (NASA-TLX). From the NASA-TLX scale we selected four subscales (mental demand, temporal demand, effort and frustration) assessing subjective workload. It was administered before the break (T2) and at the end of the class (T4). Details about this questionnaire are described by Hart and Staveland (1988).

### 2.4 Statistical analysis

Data were analyzed with the statistical program SPSS version 26.0 (IBM, NY, USA). Normality of the data was confirmed using the Shapiro-Willk test. Demographic characteristics were analyzed using descriptive statistics. The results of the VAS scales were analyzed using a repeated measures ANOVA. A post-hoc Bonferroni test was conducted to test for significant differences between time points. Paired samples t-test was used to analyze the results of NASA-TLX scale. To examine the relationship between sleep duration and mental fatigue, a correlation analysis was conducted using Pearson’s correlation coefficient. Significance was set at *p* < 0.05.

## 3 Results

In total we collected 147 individual and complete data sets from our 27 participants (spread over 8 classes).

**Figure 1:**
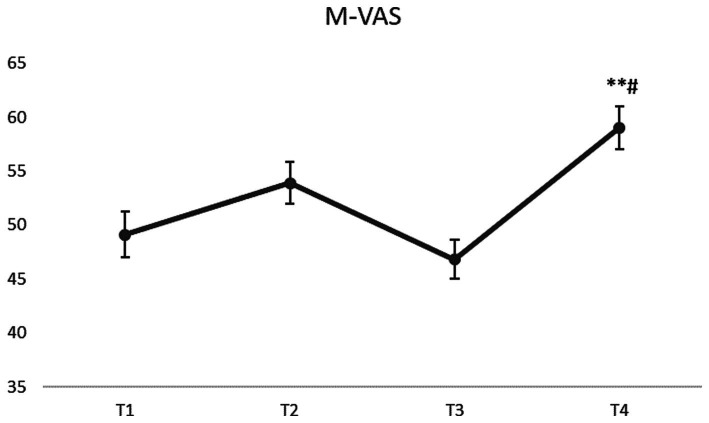
M-VAS scores over time. Results are presented as mean ± SEM. * indicates a significant difference *p* < 0.05 between T1 and T2. § indicates a significant difference *p* < 0.001 between T2 and T3. ** indicates a significant difference *p* < 0.001 between T1 and T4. # indicates a significant difference *p* < 0.001 between T3 and T4.

A significant main effect of time was found for M-VAS (*F*(1,142) = 1,041, *p* < 0.001). The initial M-VAS score at T1 was 49.11 ± 25.64, and it showed a significant increase to 53.87 ± 23.29 at T2 (*p* = 0.022). At T3, after the break, the M-VAS score significantly declined to 46.81 ± 21.91 (*p* < 0.001). After the second part of the class, displayed as T4, the M-VAS score (59.01 ± 23.38) was significantly higher compared to T1 and T3 (*p* < 0.001). Figure 1 graphically presents the results of mean scores ± standard error of the mean (SEM) over time with flagged significance.

Furthermore, we also found a significant correlation (*r* = − 0.326; *p* < 0.001) between hours of sleep the night before the class and the level of subjective mental fatigue at the start of the class (T1). The correlation coefficient indicates a moderate negative relationship between these two variables.

**Figure 2:**
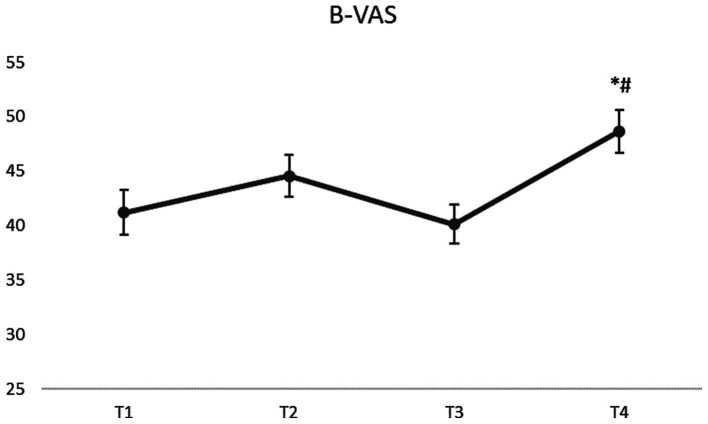
B-VAS scores over time. Results are presented as mean ± SEM. * indicates a significant difference *p* < 0.01 between T1 and T4. # indicates a significant difference *p* < 0.001 between T3 and T4.

A significant main effect of time was found for B-VAS (*F*(1,143) = 839, *p* < 0.001). The B-VAS score at T1 was 41.21 ± 24.76 and it did not significantly change at T2 (44.56 ± 22.87) (*p* > 0.05). After the break (at T3) the B-VAS score declined to 40.12 ± 21.47 (*p* > 0.05) and after the second part of the class (T4) it significantly increased to 48.65 ± 23.70 (*p* < 0.01 between T1 and T4 and *p* < 0.001 between T3 and T4). Figure 2 visually displays the mean scores ± SEM over time, highlighting significant changes.

**Figure 3:**
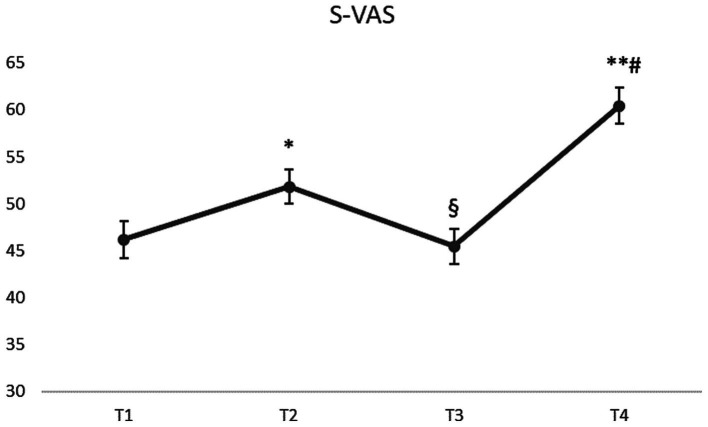
S-VAS scores over time. Results are presented as mean ± SEM. * indicates a significant difference *p* < 0.05 between T1 and T2. § indicates a significant difference *p* < 0.001 between T2 and T3. ** indicates a significant difference *p* < 0.001 between T1 and T4. # indicates a significant difference *p* < 0.001 between T3 and T4.

A significant main effect of time was found for S-VAS (F(1,143) = 1,120, *p* < 0.001). The S-VAS score at T1 was 46.18 ± 23.68 and it significantly increased to 51.83 ± 22.27 at T2 (*p* = 0.017). From T2 to T3 it significantly declined to 45.45 ± 22.77 (*p* < 0.001). Furthermore, at T4 the S-VAS score (60.44 ± 23.57) was significantly higher compared to T1 and T3 (*p* < 0.001). Figure 3 visually presents the mean scores ± SEM over time, significant changes were indicated.

Table 1 presents the results of the NASA-TLX subscales. NASA-TLX scores at T2 (before the break) and at T4 (end of the class) reveal notable variations. Mental load at T2 exhibited a mean score of 34.29 ± 17.01, significantly lower than the corresponding T4 score of 42.19 ± 19.39 (*p* < 0.001). Temporal load at T2 and Temporal load at T4 demonstrated comparable mean scores of 47.07 ± 16.36 and 47.16 ± 16.79, respectively, with no significant difference (*p* = 0.951). Effort at T2 and Effort at T4 displayed mean scores of 41.62 ± 22.59 and 46.72 ± 23.14, respectively, showing a significant increase (*p* = 0.002). Frustration at T2 had a mean score of 40.98 ± 22.98, which increased to 45.19 ± 24.03 at T4, with a significant difference (*p* = 0.027).

**Table 1:** NASA-TLX scores at T2 (before the break) and at T4 (end of the class).

	*N*	Mean ± SD	*p*
Mental load_T2	147	34.29 ± 17.01	<0.001
Mental load_T4	147	42.19 ± 19.39	
Temporal load_T2	147	47.07 ± 16.36	0.951
Temporal load_T4	147	47.16 ± 16.79	
Effort_T2	147	41.62 ± 22.59	0.002
Effort_T4	147	46.72 ± 23.14	
Frustration_T2	147	40.98 ± 22.98	0.027
Frustration_T4	147	45.19 ± 24.03	

## 4 Discussion

The current study aimed to evaluate the MF, boredom and sleepiness perceptions of Slovenian university students during a 4-h in-person (theoretical) class interspersed by a single 20 min break. As secondary objectives we aimed to correlate the feelings of MF at the start of the class with the hours of sleep the night before the class, and we wanted to determine the load that each part of the class (before and after the break) imposed on the students. The main outcomes indicate that throughout the class the MF, boredom and sleepiness were altered significantly. As expected, the break in the middle of the 4 h class reduced these perceptions significantly. Furthermore, we demonstrated that the hours of sleep the night before the class correlated with the feelings of MF at the start of the class.

Although the initial M-VAS scores were rather high, the M-VAS scores in our study were still able to demonstrate a significant increase in MF after each part of the class (T2 and T4 compared to T1) and a subsequent decrease following the break (T3). These findings suggest that the cognitive demands imposed by the class contributed to the development of MF among the students. This was also confirmed by the increased mental load reported from the NASA-TLX subscale on mental load. Indeed, our study further substantiates previous studies that identified time on task as an important contributor to the build-up of MF (Lim et al., 2010; Van Cutsem et al., 2022). One way to alleviate these negative effects of sustaining attention for prolonged periods of time is through taking one or more breaks. These allow to – at least partially – retrieve mental resources, to reorient to task goals and to adjust one’s effort allocation strategy (Qi et al., 2020). This was also confirmed in our study. We observed a temporary decline in subjective MF after the 20 min break, which indicates that the short rest interval provided some relief from this subjective experience of fatigue. This aligns nicely with the study of Qi et al. (2020) in which a mid-task break resulted in a recovery of behavioral performance in the second half of the task. The authors attributed this effect of the mid-task break to – among others - improvements in the temporal global efficiency, suggesting a general effect of a mid-task break in maintaining global efficiency of temporal information processing. Previous research by Sievertsen et al. (2016) has also demonstrated the beneficial effect of taking a 20–30 min break on mental performance in a student population. On the other hand, Brazaitis and Satas (2023) simulated 7 h office-like computer work and studied the effects of repetitive short breaks (10 min) every 50 min. The authors reported that these regular breaks were unable to prevent mental exhaustion or the cognitive impairments that coincided with this exhaustion (Brazaitis and Satas, 2023). Our findings also align with previous research by McCabe et al. (2023), who found a correlation between MF and the number and duration of academic classes. In their study, a positive correlation was observed, suggesting that increased MF was associated with both a higher number of academic classes and prolonged class durations. This implies that students experiencing a greater MF tended to participate in a larger volume of academic sessions or endured lengthier classes. The identified correlation underscores the potential impact of the academic schedule on students’ mental well-being and points toward the need for strategic interventions to address MF in the context of academic engagement. Thus, our study adds further support to the notion that academic theoretical classes induce MF, with longer class durations exacerbating these feelings.

Furthermore, a correlation analysis in our study revealed that those students who got fewer hours of sleep the night before the class had significantly higher level of subjective MF at the start of the class (at T1). It was previously demonstrated that inadequate sleep and even insomnia are prevalent among students, primarily attributed to factors such as stress related to academic performance, attendance, relationships with teachers and peers, and the struggle to balance workload and free time (Bauducco et al., 2022). The literature on single night sleep deprivation reports that less than 6 hours of sleep causes higher sleepiness, lower alertness and reduced neurobehavioral performance (Dawson and McCulloch, 2005). Inadequate sleep and general fatigue have also been identified as significant factors contributing to decreased academic engagement (Zhuang et al., 2023). Thus, the negative correlation found in our study suggests that insufficient sleep may contribute to higher levels of MF at the start of the class. These findings underscore the importance of prioritizing sufficient sleep to optimize cognitive functioning and mitigate the onset of MF during academic activities. They align with previous research highlighting the detrimental effects of sleep deprivation on cognitive performance and subjective experiences of fatigue (Belenky et al., 2003; Halbach et al., 2003). Students should be encouraged to recognize the impact of sleep on their well-being and academic performance, promoting healthy sleep habits as a crucial component of their overall cognitive functioning.

A boredom effect was only detected after the second part of the class (at T4; compared to T1 and T3). This increase in boredom may be attributed to various factors, including prolonged exposure to the same learning material, decreased engagement over time, and potential over- or under-challenging of students (Krannich et al., 2019). Additionally, the lecturing style of the teacher in the second part of the class might differ, as fatigue and a decline in enthusiasm could affect their interaction with students. Supporting these findings, Pekrun et al. (2010, 2011) reported that boredom is a common academic feeling among university students, with 42% of students reporting feelings of boredom during class. Similarly, Daniels (2009), found that first year university students experienced boredom in 40% of all academic classes. These findings indicate the need for education professionals to identify successful strategies to alleviate boredom among students in academic settings. Further research is warranted to explore the underlying factors contributing to increased boredom during prolonged academic sessions.

The findings from the assessment of sleepiness provide important insights into the fluctuation of sleepiness levels during the class session. At the beginning of the class (T1), the average S-VAS score was 46.18 ± 23.68, indicating a moderate level of sleepiness among the students. This could be influenced by factors such as students’ pre-class activities, previous cognitive demands, the timing of their schedule, or their sleeping patterns. For example, some students may have had classes in the morning or engaged in other mentally or physically demanding tasks prior to the class. Williams and Shapiro (2018) demonstrated that performance of two different students in the same class can significantly differ solely due to their prior schedules. In our study there was a significant increase in sleepiness levels following the completion of the first part of the class (T2). This suggests that the initial cognitive demands and the extended duration of the class contributed to an additional rise in sleepiness among the students. It is important to note that after the 20 min break (T3), there was a significant decline in sleepiness levels. This temporary reduction can be attributed to the beneficial effects of the break, allowing the students to rest and recharge to some extent. The importance of taking breaks from sitting was also noted in a study from Hosteng et al. (2019), which explored the relation between uninterrupted sitting and perceived levels of physical discomfort and sleepiness among college students. Notably, the participants reported significant impairments in discomfort after 75 min and sleepiness after 15 min of uninterrupted sitting (Hosteng et al., 2019).

While our study provides valuable insights into the perceptions of mental fatigue, boredom, and sleepiness among university students during in-person theoretical classes, there are certain limitations to consider. First, the study focused on a specific cohort of first-year physiotherapy students, which may limit the generalizability of the findings to other academic disciplines or student populations. Future research should include a more diverse sample to enhance the external validity of the results. Additionally, the study relied only on subjective self-report measures to assess MF, boredom, and sleepiness, which may be influenced by individual interpretation and response biases. Objective measures, such as neurophysiological or performance-based assessments, could complement the subjective data and provide a more comprehensive understanding of students’ cognitive states. Lastly, our study did not investigate specific factors contributing to MF, boredom, or sleepiness during the class. Future research could explore potential factors such as class content, teaching methods, classroom environment, or individual characteristics that may influence students’ subjective experiences (Russell et al., 2019). Future studies should also place emphasis on investigating the effectiveness of various strategies, such as the integration of active breaks or the implementation of more frequent short breaks, in alleviating these negative feelings.

This study highlighted the impact of prolonged in-person theoretical classes on university students’ perceptions of MF, boredom, and sleepiness. The findings suggest that these subjective experiences fluctuate throughout the class. A 20 min break appears to temporarily alleviate these subjective states, but they tend to reemerge as the class progresses. Sleep duration the night before the class also influences the level of MF at the beginning of the class. This study emphasizes the importance of adequate sleep, effective breaks, and strategies to manage cognitive workload in optimizing students’ cognitive well-being. Further research is needed to better understand the underlying factors and develop targeted interventions to support students’ cognitive functioning and well-being during prolonged academic sessions.

## Data availability statement

The raw data supporting the conclusions of this article will be made available by the authors, without undue reservation.

## Ethics statement

The studies involving humans were approved by Ethics Committee for Research Involving Human Subjects, Faculty of Health Sciences, University of Novo mesto (UNM 141/2023). The studies were conducted in accordance with the local legislation and institutional requirements. The participants provided their written informed consent to participate in this study.

## Author contributions

PK: Conceptualization, Investigation, Writing – original draft. BR: Conceptualization, Writing – review & editing. ŠB: Conceptualization, Investigation, Writing – review & editing.
